# Unveiling the therapeutic benefits of black chokeberry (*Aronia melanocarpa*) in alleviating hyperuricemia in mice

**DOI:** 10.3389/fnut.2025.1556527

**Published:** 2025-05-14

**Authors:** Chin-Yuan Liu, Wen-Yu Liu, Yeu-Ching Shi, She-Ching Wu

**Affiliations:** ^1^Department of Food Sciences, National Chiayi University, Chiayi, Taiwan; ^2^Department of Food Safety/Hygiene and Risk Management, College of Medicine, National Cheng Kung University, Tainan, Taiwan

**Keywords:** adenosine deaminase, black chokeberry, hyperuricemia, uric acid, xanthine oxidase

## Abstract

**Background:**

Hyperuricemia not only increases the risk of cardiovascular diseases such as dyslipidemia, hypertension, coronary artery disease, obesity, metabolic syndrome, and type-2 diabetes, but also severely impacts kidney function, potentially leading to acute kidney injury and chronic kidney disease.

**Methods:**

This study aims to investigate the health benefits of black chokeberry (*Aronia melanocarpa*) on hyperuricemic mice induced by oxonic acid.

**Result:**

The experimental results showed that black chokeberry had no significant toxic or negative effects in mice. The measurement of uric acid (UA) indicated that black chokeberry suppressed the UA levels. Additionally, the xanthine oxidase activity in the high-dose group was significantly decreased, along with reductions in serum urea nitrogen and creatinine levels. Black chokeberry effectively increased the glutathione levels in hyperuricemic mice and reduced malondialdehyde levels, as well as significantly inhibiting adenosine deaminase activity.

**Conclusion:**

Its efficacy is comparable to that of the marketed drug allopurinol, underscoring the potential of black chokeberry as a functional product for uric acid reduction.

## 1 Introduction

Hyperuricemia refers to an excessively high concentration of uric acid (UA) in the serum. Typically, hyperuricemia is diagnosed when serum UA levels exceed 7 mg/dL in men and 6 mg/mL in women. The global prevalence of hyperuricemia is approximately 19.37%, with higher incidences observed in men and postmenopausal women (1). As the condition progresses, it may develop into gouty arthritis, kidney stones, and cause renal damage (2).

UA is the final product of purine metabolism, with most serum UA originating from endogenous purines, and about one-third coming from dietary sources. Hyperuricemia is caused by excessive production of UA and insufficient renal excretion. Therefore, current medications for lowering UA primarily work by promoting UA excretion and inhibiting xanthine oxidase (XO) (3). However, these drugs are often associated with side effects such as allergies, diarrhea, hepatotoxicity, and nephrotoxicity (4, 5). Hence, there is a need for alternative safe and effective treatments for hyperuricemia.

*Aronia melanocarpa*, commonly known as black chokeberry, is a berry rich in polyphenolic compounds and flavonoids. Its main active components include chlorogenic acid, neochlorogenic acid, anthocyanins, proanthocyanidins, and quercetin derivatives. Numerous studies have confirmed its health benefits, including antioxidant, anti-inflammatory, antimicrobial, antihypertensive, lipid-lowering, antidiabetic, hepatoprotective, and neuroprotective effects (6). Previous studies have shown that black chokeberry can improve serum UA levels, reduce xanthine oxidase activity, and inhibit inflammation associated with acute gout in rat models (7). Additionally, black chokeberry has been shown to alleviate kidney damage by reducing the expression of pro-inflammatory factors, oxidative stress, lipid peroxidation, and apoptosis, thereby improving renal function (8).

This study used domestically sourced black chokeberry to evaluate serum UA, blood urea nitrogen (BUN), and creatinine (CRE) levels in mice, assessing kidney function. It also measured xanthine oxidase, glutathione (GSH), malondialdehyde (MDA), and adenosine deaminase (ADA) activity to assess liver function. The UA-lowering effects of black chokeberry were further explored in hyperuricemic mice induced by oxonic acid potassium salt (OA). Black chokeberry demonstrated potential in inhibiting UA production, reducing BUN, and lowering creatinine levels, while also inhibiting the activity of xanthine oxidase and adenosine deaminase. These results suggest that black chokeberry may improve both kidney and liver function in mice and holds promise as a functional product for lowering UA.

## 2 Methods

### 2.1 Sample preparation

After juicing for black chokeberries, the juice was filtered through a 40-mesh sieve and then vacuum filtered to remove the skin and pulp. The filtrate was then freeze-dried using a vacuum freeze dryer, and the resulting dried powder constituted the black chokeberry. This powder was stored at −20°C for subsequent experiments.

### 2.2 Animals

This study was approved by the Laboratory Animal Care and Use Committee of National Chiayi University (Approval No. 112021). Male ICR mice, 5 weeks old, were purchased from LASCO Biotechnology Co., Ltd. to establish an oxonic acid potassium salt (OA)-induced hyperuricemia model by ip. After acclimatizing the mice for 1 week, they were randomly divided into six groups, each with six mice: blank, OA (250 mg/kg bw/day) induction by intraperitoneal injections, OA + allopurinol (10 mg/kg bw/day), OA + low-dosage sample (450 mg/kg bw/day), OA + medium-dosage sample (900 mg/kg bw/day), and OA + high-dosage sample (1800 mg/kg bw/day). The experiment lasted for 1 week. After the experiment, blood was collected via cardiac puncture for biochemical analysis, and the liver and kidneys were dissected. The organs were rinsed with saline, weighed, wrapped in aluminum foil, and rapidly frozen in liquid nitrogen, then stored at-80°C for further analysis.

### 2.3 Serum preparation

Place the collected blood in a tube without anticoagulant and allow it to clot at room temperature for 1 h, and then centrifugation was carried out at 3,000 rpm for 10 min at 4°C to separate the serum and stored at −80°C.

### 2.4 Liver homogenate preparation

Liver (0.4 g) was homogenated with PBS, and the solution was centrifuged at 5,000 rpm for 10 min at 4°C, the supernatant was collected and stored at −80°C.

### 2.5 Measurement of blood urea nitrogen

Analyze using the Urea Enzymatic Kinetic Method commercial kit (RANDOX). Add 10 μL of serum and the standard solution to 1 mL of the reaction reagent containing α-oxoglutarate, ADP, urease, GLDH, NADH, and Tris buffer (pH 7.6) for incubation at 37°C for 30 s, then the absorbance at 340 nm was measured. Continue the reaction for another 60 s and measure the absorbance at 340 nm again.

The calculation formula: BUN = Standard concentration × ΔA_sample, 340 nm_/ΔA_standard, 340 nm_.

### 2.6 Measurement of serum creatinine content

Analyze using the Creatinine commercial kit (RANDOX). Add 100 μL of serum and the standard solution to 1 mL of the reaction reagent containing 35 μmol/L picric acid and 0.32 mol/L sodium hydroxide. Measure the absorbance at 492 nm after 30 s and again after 150 s.

The calculation formula: CRE = Standard concentration × ΔA_sample, 492 nm_/ΔA_standard, 492 nm_.

### 2.7 Measurement of serum uric acid content

Analyze using the Uric Acid commercial kit (RANDOX). Add 20 μL of serum and the uric acid standard solution to 1 mL of the reaction reagent containing HEPES buffer, 3,5-dichloro-2-hydroxybenzenesulfonic acid, 4-aminophenazone, uricase, and peroxidase. Mix thoroughly and incubate in a water bath at 37°C for 5 min. Measure the absorbance at 520 nm.

The calculation formula: UA = Standard concentration × (A_sample, 520 nm_/A_standard, 520 nm_).

### 2.8 Measurement of xanthine oxidase activity

The activity of xanthine oxidase in the liver is measured based on the conversion of xanthine to uric acid (9). Adding 100 μL of liver homogenate and the uric acid standard solution to 4.9 mL of the reaction reagent containing 50 μM xanthine and 5 mM EDTA. Incubate at 37°C for 30 min, then add 500 μL of 0.58 M HCl to terminate the reaction. Measure the absorbance at a wavelength of 290 nm.

### 2.9 Measurement of glutathione content

Liver homogenate (100 μL) to 100 μL of 5% TCA solution and incubate on ice for 5 min. Centrifugating at 12,000 rpm for 15 min, and collect 150 μL of the supernatant. Add this to 750 μL of Tris/EDTA solution (pH 8.2) and mix thoroughly. Then, add 37.5 μL of 5,5′-DithioBis-(2-Nitrobenzoic Acid) (DTNB) solution, incubate for 5 min, and measure the absorbance at 412 nm (10).

### 2.10 Measurement of malondialdehyde content

Liver tissue homogenate (40 μL) and TEP standard to 120 μL of deionized water and 40 μL of 8.1% SDS solution. Mix thoroughly and incubate at room temperature for 5 min. Then, add 300 μL of 20% ethanolic acid (pH 3.5) and 300 μL of 0.8% TBA reagent, and incubate at 95°C for 1 h. After cooling, centrifuge at 4,000 rpm for 10 min and measure the absorbance of the supernatant at 532 nm (11).

### 2.11 Measurement of adenosine deaminase activity

Analyze using the Elabscience commercial kit (Metabolism Assay). Add 10 μL of liver homogenate and the standard solution to 180 μL of Reagent-1 and 90 μL of Reagent-2. Incubate at 37°C for 7 min, then measure the absorbance at 550 nm. After this, continue the reaction at 37°C for another 10 min and measure the absorbance again at 550 nm.

### 2.12 Statistical methods

The results of this experiment are expressed as mean ± standard deviation (S.D.). Data analysis was conducted using SPSS (Statistical Product and Service Solutions 22.0). One-way ANOVA was used to compare differences between groups, followed by Duncan’s multiple range test for significance comparisons. A *p*-value of < 0.05 indicates a statistically significant difference in the data.

## 3 Results and discussion

### 3.1 Body weight, food intake, water intake, and organ weight

If toxic substances enter the experimental animals, they can affect their physiological condition or metabolic processes, leading to significant changes in body weight. To observe whether intraperitoneal injection or feeding of drugs and samples causes toxicity in animals, changes in body weight can be used as a preliminary indicator of potential poisoning (12).

Table 1 presents the body weight changes in hyperuricemia-induced mice. No significant differences in body weight were observed among the groups, except for the positive control group, where drug treatment likely improved physiological status, leading to better dietary intake and increased weight. Daily food and water intake were monitored, with an average consumption of 5.3 g and 9.1 g per mouse, respectively. In conclusion, black chokeberry supplementation did not cause toxicity or significantly affect body weight, food and water intake in hyperuricemic mice induced by intraperitoneal OA injection.

**Table 1:** Changes in body weight, dietary intake, and water intake of OA-induced hyperuricemia mice.

Groups	Body weight (g)		Food intake (g)		Water intake (g)	
	Day-1	Day-7	Day-1	Day-7	Day-1	Day-7
Blank	27.78 ± 2.9^a^	29.98 ± 2.37^a^	6.8	6.27	7	12
OA	27.90 ± 1.64^a^	28.48 ± 2.06^a^	5.22	5.67	8	9
OA + allopurinol	28.53 ± 1.02^b^	30.62 ± 1.83^a^	5.66	5.96	10	11
OA + low dosage	27.78 ± 1.39^a^	27.7 ± 4.29^a^	5.61	4.74	8	12
OA + medium dosage	27.70 ± 0.95^a^	28.17 ± 1.33^a^	4.98	4.58	8	6
OA + high dosage	27.45 ± 1.24^a^	28.51 ± 3.10^a^	5.71	4.52	8	11

Each value is expressed as mean ± S.D. (*n* = 6). Value in row with the different superscripts are significantly different (*p* < 0.05).

### 3.2 Serum BUN and CRE

BUN is a nitrogenous molecule produced in the liver and excreted by the kidneys, commonly used to assess kidney function (13). This experiment evaluates whether different doses of black chokeberry can reduce BUN levels in hyperuricemic mice (Table 2). The OA-induced group exhibited BUN level (13.51 mmol/L) compared to the control group (4.54 mmol/L). In the low-, medium-, and high-dosage groups, the levels of BUN were reduced to 10.21, 9.56, and 4.86 mmol/L, reflecting reductions of 24.43, 29.24, and 64.03%, respectively, compared to the OA-induced group. Notably, the high-dosage group showed a significant reduction, with levels falling below those of the allopurinol-treated group (6.81 mmol/L). Moreover, the high-dosage group outperformed the OA-induced mice treated with allopurinol, showing no significant difference compared to the control group. These findings suggested that high-dosage sample treatment in hyperuricemic mice lowered BUN.

**Table 2:** Effect of black chokeberry on blood urea nitrogen and creatinine levels in hyperuricemic mice.

Groups	BUN (mmol/L)	CRE (μmol/L)
Blank	4.54 ± 1.14^c^	9.01 ± 0.98^d^
OA	13.51 ± 3.57^a^	38.87 ± 1.69^a^
OA + allopurinol	6.81 ± 1.63bc	11.83 ± 2.93^cd^
OA + low dosage	10.21 ± 2.26^ab^	23.66 ± 4.78^b^
OA + medium dosage	9.56 ± 1.79^ab^	15.21 ± 1.69^c^
OA + high dosage	4.86 ± 2.35^c^	9.58 ± 0.98^d^

Each value is expressed as mean ± S.D. (*n* = 6). Value in row with the different superscripts are significantly different (*p* < 0.05).

Table 2 presents the effects of black chokeberry on CRE levels in hyperuricemic mice. The negative control group exhibited CRE level (38.87 μmol/L), which approximately three times higher than that of the control group (9.01 μmol/L). In the low-, medium-, and high-dose groups (black chokeberry), the CRE levels were 23.66, 15.21, and 9.58 μmol/L, respectively, showing a dose-dependent reduction of 39.13, 60.87, and 75.35%. The CRE level in the high-dose group was comparable to that of the control group and not significantly different from the positive control group, suggesting that high-dose black chokeberry treatment achieved similar reductions in CRE as drug treatment and brought levels close to those of uninduced mice.

Hyperuricemia induced by OA can lead to nitric oxide-related endothelial dysfunction, affecting the kidney’s ability to transport urate and other organic ions, thereby worsening renal dysfunction and damage (14). Cherry has known for its immunomodulatory properties, can enhance the immune system through various mechanisms (14, 15). These findings suggest that high-dose wild cherry effectively reduces CRE levels in hyperuricemic mice, highlighting potential of black chokeberry used as a natural kidney-protective agent.

### 3.3 Serum uric acid and xanthine oxidase activity

Uricase is an enzyme in mammals that converts uric acid to allantoin. In animal studies, oxonic acid, a commonly used uricase inhibitor, induces hyperuricemia by raising serum uric acid levels. It is cost-effective, fast-acting, and widely used in preliminary research to evaluate the uric acid-lowering effects of new treatments for hyperuricemia (16, 17). Clinically, hyperuricemia is treated with drugs that either promote uric acid excretion (such as probenecid, benzbromarone, and sulfinpyrazone) or inhibit uric acid production (such as allopurinol and febuxostat). However, these medications are associated with side effects, including renal toxicity, liver damage, and an increased risk of cardiovascular disease (18, 19). This has led to growing interest in natural products rich in phytochemicals as alternative treatments for hyperuricemia, offering potential uric acid-lowering effects with fewer side effects.

**Figure 1:**
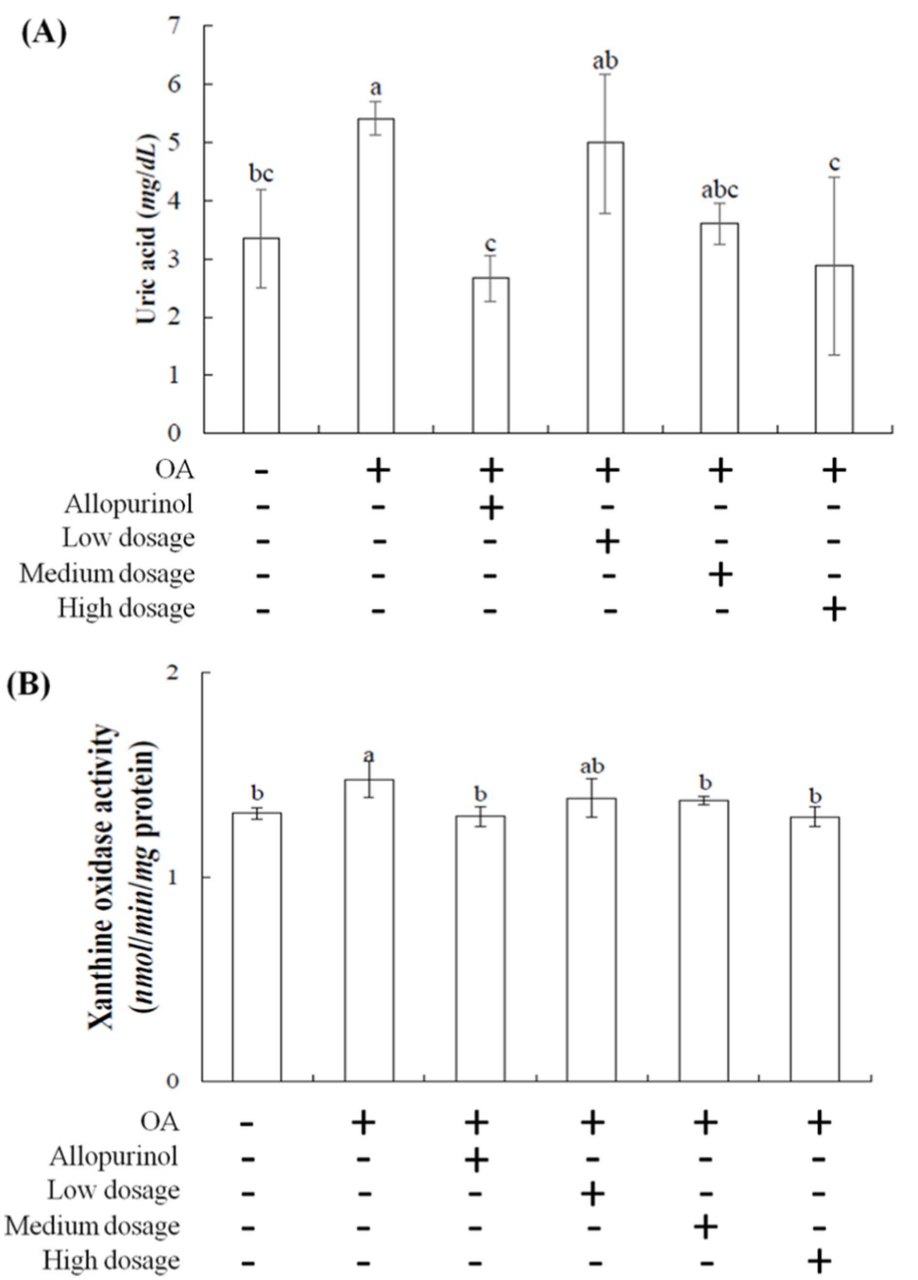
Effect of black chokeberry on serum **(A)** uric acid levels and **(B)** liver xanthine oxidase activity in hyperuricemicmice. Each value is expressed as mean ± S.D. (*n* = 6). Value in row with the different superscripts are significantly different (*p* < 0.05).

Figure 1A illustrates the effects of black chokeberry on serum uric acid level in hyperuricemic mice. One week after OA injection, the serum uric acid level in the OA-induced mice was rose to 5.40 mg/dL compared to the control group (3.35 mg/dL). In contrast, the OA-induced mice treated with allopurinol showed a reduction to 2.66 mg/dL, below the level of the control group. In the low-, medium-, and high-dosage groups, serum uric acid level was decreased in a dose-dependent manner, reaching 4.98, 3.61, and 2.87 mg/dL, respectively. The high-dosage group demonstrated the most pronounced effect, with results comparable to the allopurinol group.

Patients using allopurinol for hyperuricemia are at an increased risk of requiring dialysis (10). This study showed the effects of black chokeberry treating hyperuricemia in mice are comparable to those of the synthetic drug allopurinol. The results suggested that black chokeberry has notable uric acid-lowering efficacy, likely due to its phenolic acid components (19, 20), making it a promising candidate for development into health products targeting uric acid-related conditions.

Xanthine oxidase is the primary enzyme for purine metabolism in the liver (21, 22). It oxidizes hypoxanthine to xanthine and then xanthine to uric acid, generating superoxide anions (O₂^−^) and peroxides, which increase cellular oxidative stress. Excess uric acid in the body can lead to hyperuricemia and gout, caused by the crystallization of uric acid in joints and surrounding tissues (23). Increased intake of high-purine foods can lead to overactivity of xanthine oxidase, raising the prevalence of hyperuricemia. Allopurinol and febuxostat are two xanthine oxidase inhibitors but can have significant side effects (24).

Developing natural compounds as xanthine oxidase inhibitors is essential. Figure 1B showed the effects of black chokeberry on xanthine oxidase activity in the livers of hyperuricemic mice. No significant differences were observed in xanthine oxidase activity among the low-, medium-, and high-dosage groups compared to the control group. However, both the high-dosage and allopurinol treatment showed significant reductions compared to the OA-induced group. These findings suggest that high-dosage sample effectively reduces xanthine oxidase activity, with similar efficacy to allopurinol, thereby lowering uric acid level.

### 3.4 GSH and MDA levels in hyperuricemic mice

Glutathione (GSH) is present in most mammals, synthesized from glutamate and cysteine, and plays a crucial role as a key antioxidant. It helps generate iron–sulfur proteins and neutralizes reactive oxygen species (ROS), controlling redox states (25). High concentrations of GSH protect the liver from oxidative stress (26). Research by Zhang et al. (27) shows that CCl₄-induced liver damage depletes GSH due to its conversion to oxidized glutathione during free radical scavenging. Notably, anthocyanins in wild cherries may help maintain liver GSH levels.

Table 3 showed the effects of black chokeberry on GSH content in the livers of hyperuricemic mice. The GSH levels in the control, OA induction, allopurinoll, low-dosage, medium-dosage, and high-dosage treated-groups were 2.82, 1.90, 3.18, 3.29, 3.47, and 3.88 μmol/mg protein, respectively. Black chokeberry treatment resulted in an upward trend in GSH content. These results suggested that high doses of black chokeberry may boost GSH levels, helping to prevent or mitigate liver damage caused by oxidative stress, thereby reducing hepatic oxidative stress injury.

**Table 3:** The GSH and MDA contents in the liver of OA-induced hyperuricemic mice.

Groups	GSH (umol/mg protein)	MDA (umol/mg protein)
Blank	2.82 ± 0.34^ab^	1.12 ± 0.09^bc^
OA	1.9 ± 0.67^c^	1.29 ± 0.09^a^
OA + allopurinol	3.18 ± 1.16^ab^	1.19 ± 0.1^b^
OA + low dosage	3.29 ± 1.34^ab^	1.18 ± 0.11^b^
OA + medium dosage	3.47 ± 1.24^b^	1.13 ± 0.13^bc^
OA + high dosage	3.88 ± 1.32^b^	1.07 ± 0.11^c^

Each value is expressed as mean ± S.D. (*n* = 6). Value in row with the different superscripts are significantly different (*p* < 0.05).

Malondialdehyde (MDA) is widely used as an indicator of lipid peroxidation and reflects oxidative stress in cells, particularly from reactive oxygen species (ROS) (28). MDA can disrupt lipid membrane structures, leading to severe cellular damage (29). A study indicated that flavonoids in black chokeberry can reduce CCl₄-induced lipid peroxidation in the liver (30). Table 3 presents the protection of black chokeberry on MDA levels in the livers of OA-induced hyperuricemic mice. The MDA concentrations for the control, OA induction, allopurinol, low-dosage, medium-dosage, and high-dosage groups being 1.09, 1.31, 1.13, 1.14, 1.09, and 1.01 μmol/mg protein, respectively. Notably, there was a significant difference between the high-dose and positive control groups. Elseweidy et al. (31) showed that allopurinol treatment could modulate liver lipid metabolism and reduce MDA levels in fructose-induced non-alcoholic fatty liver models. These findings suggest that high-dosage black chokeberry can significantly lower liver MDA levels, thereby mitigating oxidative stress-related damage.

### 3.5 Adenosine deaminase activity in the OA-induced hyperuricemic mice

**Figure 2:**
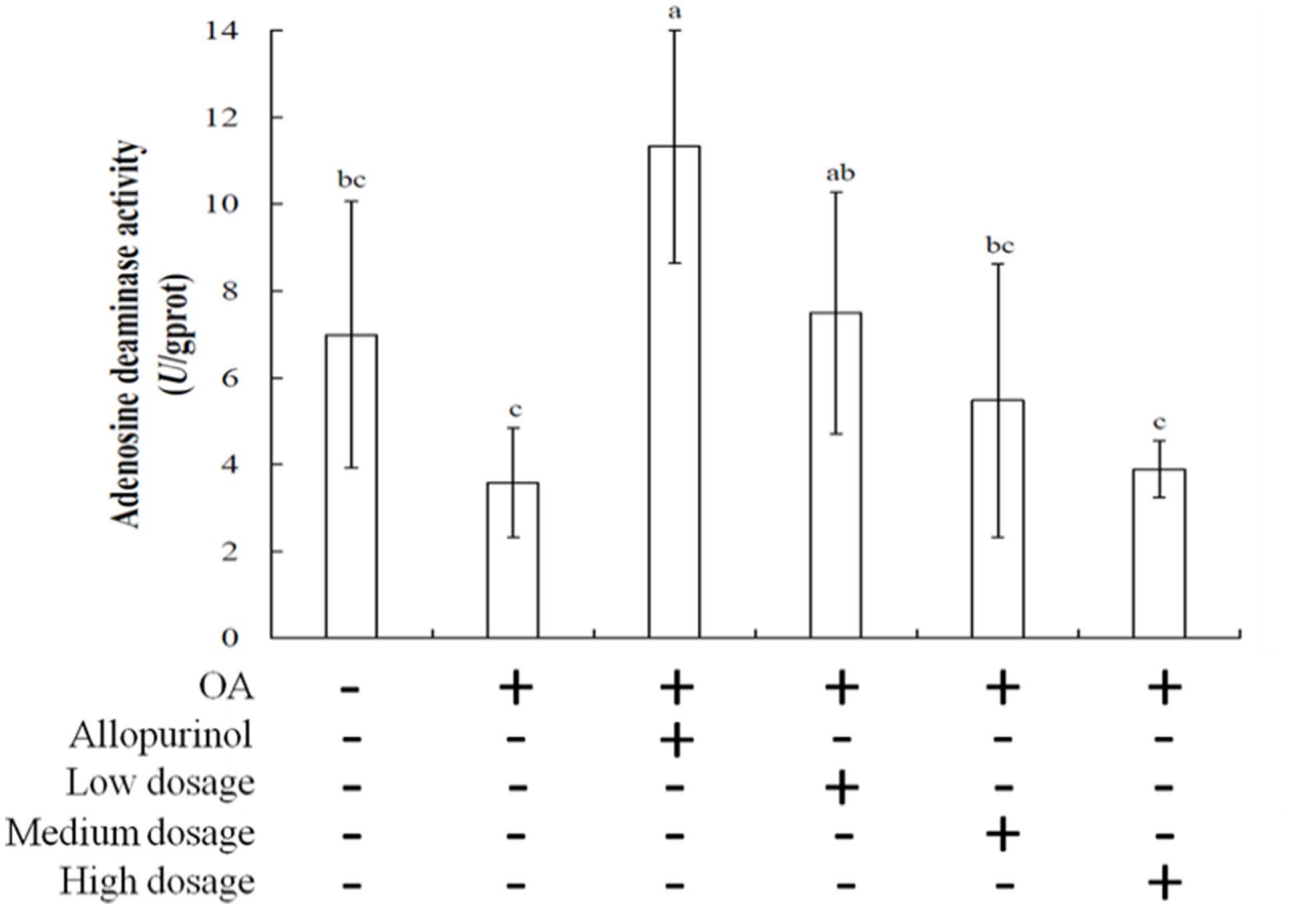
Effect of black chokeberry on liver adenosine deaminase activity in hyperuricemic mice. Each value is expressed as mean ± S.D. (*n* = 6). Value in row with the different superscripts are significantly different (*p* < 0.05).

Adenosine deaminase (ADA) is a hydrolytic enzyme involved in purine metabolism, converting adenosine and 2′-deoxyadenosine into inosine and 2′-deoxyinosine, respectively (32). ADA is found in various organisms, including plants, bacteria, and animals (33). Inhibition of ADA can modulate inflammation by increasing adenosine levels in damaged tissues, thereby reducing inflammatory responses. Figure 2 illustrated the effects of wild cherry on ADA activity in the livers of hyperuricemic mice. The ADA activities for the control, OA induction, allopurinol, low-dosge, medium-dosage, and high-dosage groups were 6.99, 11.33, 3.58, 7.50, 5.47, and 3.89 μmol/mg protein, respectively. Notably, the high-dosage group showed lower ADA activity than the control group. Due to ADA activity increases with rising uric acid level; therefore, administering high-dosage of black chokeberry can lower uric acid levels and inhibit ADA activity, subsequently reducing inflammation.

Gout is an inflammatory disease caused by excessive uric acid levels in the blood, leading to the formation of insoluble monosodium urate (MSU) crystals. These crystals drive neutrophil activation and infiltration into joint tissues, resulting in severe pain in the affected areas. MSU crystals mediate oxidative stress through the nucleotide-binding oligomerization domain-like receptor pyrin domain-containing 3 (NLRP3) inflammasome, which subsequently triggers interleukin-1β production, exacerbating the inflammatory response (34–36).

Xanthine oxidase is a key enzyme in the purine metabolism pathway, and clinical and experimental studies have suggested that its activity may have pro-inflammatory effects (37). Currently, various anti-gout medications are available, including nonsteroidal anti-inflammatory drugs (NSAIDs) such as allopurinol, which are commonly used as first-line treatments for acute gout. However, their use is limited by adverse effects, including gastrointestinal toxicity, renal toxicity, and gastrointestinal bleeding. Therefore, the development of more effective anti-gout arthritis drugs remains of great significance (38, 39). In recent years, we have identified the bioactive compounds and antioxidants in black chokeberry (40). These findings suggest that black chokeberry has the potential for gout prevention and management.

## 4 Conclusion

Hyperuricemia is a pathological condition characterized by an abnormal increase in serum uric acid levels due to excessive uric acid production or reduced excretion. The main pathogenic mechanisms include xanthine oxidase overactivation and phosphoribosyl pyrophosphate synthetase (PRPP synthetase) hyperactivity. For xanthine oxidase, which promotes the conversion of xanthine to uric acid, leading to excessive uric acid production, but PRPP synthetase is able to enhance purine synthesis, subsequently increasing uric acid production. Uric acid is the final product of purine metabolism, derived from both endogenous (cellular metabolism) and exogenous (dietary) purine breakdown. A high-purine diet (e.g., red meat, organ meats, and seafood), excessive fructose intake, alcohol consumption, and obesity are closely associated with the development of hyperuricemia. In this study, our results demonstrate that black chokeberry can reduce xanthine oxidase activity and uric acid production. In the future, it may serve as a potential functional ingredient for gout prevention.

## Data Availability

The raw data supporting the conclusions of this article will be made available by the authors, without undue reservation.
